# Reviewer acknowledgement 2012

**DOI:** 10.1186/1757-7241-21-6

**Published:** 2013-03-12

**Authors:** Hans Morten Lossius, Kjetil Søreide

**Affiliations:** 1 Norwegian Air Ambulance Foundation, Drøbak, Norway; 2 Stavanger University Hospital, Stavanger, Norway

## Abstract

**Contributing reviewers:**

The Editors of *Scandinavian Journal of Trauma, Resuscitation and Emergency Medicine (SJTREM)* would like to thank all our reviewers who have contributed to the journal in Volume 20 (2012).

## 

Samar Aboulsoud

Qatar

Terrence Adam

United States of America

Sara Ahmadi

United States of America

Peter Aitken

Australia

César Alameda

Spain

Yavuz Albayrak

Turkey

Gidon Almogy

Israel

Tore Amundsen

Norway

Hagen Andruszkow

Germany

Filip K. Arnberg

Sweden

Takashi Asai

Japan

M Hammad Ather

Pakistan

Matt Baker

United Kingdom

Miklosh Bala

Israel

Chad Ball

Canada

Angela Bång

Sweden

Charlotte Barfod

Denmark

Kristian Bartnes

Norway

Jonathan Benger

United Kingdom

Daniel Bergum

Norway

Joost Bierens

Netherlands

Walter Biffl

United States of America

Poul Jannik Bjerrum

Denmark

Lars Petter Bjørnsen

Norway

Conrad Arnfinn Bjørshol

Norway

Mikkel Brabrand

Denmark

Raoul Breitkreutz

Germany

Jasper Brener

United States of America

Karim Brohi

United Kingdom

Hermann Brugger

Italy

Brandon Bruns

United States of America

Pedro Caballero

Spain

Pauline Calleja

Australia

Daniel Candinas

Switzerland

Anthony Chahal

Canada

Douglas Chamberlain

United Kingdom

Sandrine Charpentier

France

Ray-Jade Chen

Taiwan

Lei Chen

United States of America

Ivy Cheng

Canada

Linda Chlan

United States of America

Erika Frischknecht Christensen

Denmark

Nicholas Clement

United Kingdom

Tim Coats

United Kingdom

Niamh Collins

Ireland

Jim Connolly

United Kingdom

Roberto Copetti

Italy

Oguz Coskun

Germany

Tobias Cronberg

Sweden

Michael Curtis

United Kingdom

Kate Curtis

Australia

Marianne Cusick

United States of America

Michel Debacker

Belgium

Trond Dehli

Norway

Francesco Della Corte

Italy

Vidyasagar Dharmapuri

United States of America

Erwin Dhondt

Belgium

Stefano Di Bartolomeo

Italy

Ahmadreza Djalali

Sweden

David Dries

United States of America

Edward Duncan

United Kingdom

Bruno Durrer

Switzerland

Ulf Ekelund

Sweden

Robert Eley

Australia

Hossein Elgafy

United States of America

Blaine Enderson

United States of America

Aristomenis K Exadaktylos

Switzerland

Sabina Fattah

Norway

Eric Feucht

United States of America

Espen Fevang

Norway

Gerry Fitzgerald

Australia

Sascha Flohe

Germany

Jakob Lundager Forberg

Sweden

Sune Forsberg

Sweden

Thomas Frauenfelder

Switzerland

Satoshi Fujita

Japan

Anna Gagliardi

Canada

Miguel Galicia

Spain

Giorgio Gambale

Italy

Jonn-Terje Geitung

Norway

Heather Gilbertson

Australia

Sven Erik Gisvold

Norway

Ernestina Gomes

Portugal

Andreas Grabinsky

United States of America

Asger Granfeldt

Denmark

Alasdair Gray

United Kingdom

Colin Grissom

United States of America

Marit Grønning

Norway

Michael Gropper

United States of America

Bjorn Gunnarsson

Iceland

Jostein S Hagemo

Norway

Ellen Merete Hagen

Norway

Peter Hallas

Denmark

Neil Hampson

United States of America

Anthony Handley

United Kingdom

Dan Hanfling

United States of America

Britt Sætre Hansen

Norway

Katherine Harding

Australia

Dietrich Hasper

Germany

Noritake Hata

Japan

Bjorn Haug

Norway

Bjørn Olav Haugen

Norway

Johan Herlitz

Sweden

Atsushi Hiraide

Japan

Magnus Hjortdahl

Norway

Corinne Hohl

Canada

Renee Holleran

United States of America

Susan Huckson

Australia

Olivier Huet

Australia

Steinar Hunskaar

Norway

Per Kristian Hyldmo

Norway

Harri Hyppölä

Finland

Toshiaki Iba

Japan

Joji Inamasu

Japan

Jan Jakobsson

Sweden

Helena Jäntti

Finland

Maria Maj Jensen

Denmark

Per Ingemar Johansson

Denmark

Gregory Jurkovich

United States of America

Annika Kaisdotter Andersson

Sweden

Jeffry Kashuk

United States of America

Niels Katballe

Denmark

Fulvio Kette

Italy

Akio Kimura

Japan

Idar Kirkeby-Garstad

Norway

Hans Kirkegaard

Denmark

Thomas Kjellström

Sweden

Lars Knudsen

Denmark

Ulf Kongsgaard

Norway

Niels Henrik Vinther Krarup

Denmark

Uwe Kreimeier

Germany

Thomas Kristiansen

Norway

Jouni Kurola

Finland

Kirk Lalwani

United States of America

Alf Inge Larsen

Norway

Jens Lauritsen

Denmark

Kugjong Lee

Korea, South

Ari Leppaniemi

Finland

Philipp Lichte

Germany

Freddy Lippert

Denmark

John Lloyd

United Kingdom

Herman Lonnée

Norway

Carsten Lott

Germany

Hsin-Min Lu

Taiwan

Christian Lund

Norway

Richard Lyon

United Kingdom

Mark Lyttle

United Kingdom

Russell Macdonald

Canada

Roderick Mackenzie

United Kingdom

Raija Malmström

Finland

Stephan Marsch

Switzerland

Suzanne Mason

United Kingdom

Eric Maury

France

Michael McCurdy

United States of America

Jason McMullan

United States of America

Carl McQueen

United Kingdom

Ari Mennander

Finland

Søren Mikkelsen

Denmark

Koenraad Monsieurs

Belgium

Joan Montaner

Spain

Damian Morgan

Australia

Ian Mosley

Australia

Veronique Moulaert

Netherlands

Nicolas Mpotos

Belgium

Brian Mucci

Uzbekistan

Pål Aksel Næss

Norway

Giuseppe Nardi

Italy

Pradeep Navsaria

South Africa

Erik W Nielsen

Norway

Leila Niemi-Murola

Finland

Martin Nordberg

Sweden

Trond Nordseth

Norway

Theresa Mariero Olasveengen

Norway

Jonathan Olshaker

United States of America

Per Örtenwall

Sweden

Adi Osman

Malaysia

Petr Ostadal

Czech Republic

Nils Petter Oveland

Norway

Peter Paal

Austria

Henrik Palm

Denmark

Cameron Palmer

Australia

Jomon Paul

United States of America

Tommaso Pellis

Italy

Gavin D Perkins

United Kingdom

Roman Pfeifer

Germany

Michael Quirke

Ireland

Marizen Ramirez

United States of America

Marius Rehn

Norway

Arne Rehnsfeldt

Sweden

Schalk Richard

Germany

Sandra Richardson

New Zealand

Kjetil G. Ringdal

Norway

Emanuel Rivers

United States of America

Derek Roberts

Canada

Leif Rognås

Denmark

Bertil Romner

Denmark

Ole Morten Ronning

Norway

Urban Safwenberg

Sweden

Ayşe San Türgay

Turkey

Michael Sand

Germany

Mårten Sandberg

Norway

James Sanger

United States of America

Gianfranco Sanson

Italy

Danielle Sartorius

Switzerland

Thomas Scalea

United States of America

Niels Schep

Netherlands

Ulf Martin Schilling

Sweden

Kari Schirmer-Mikalsen

Norway

Herbert Schöchl

Austria

David Seder

United States of America

Harald Selig

Austria

Tom Silfvast

Finland

Arne Skjold

Norway

David Sklar

United States of America

Eirik Skogvoll

Norway

Markus Skrifvars

Finland

Erik Sloth

Denmark

Kanwalpreet Sodhi

India

Stephen J M Sollid

Norway

Sasa Sopka

Germany

Eldar Søreide

Norway

Jon Arne Søreide

Norway

Signe Sovik

Norway

Philip Stahel

United States of America

Petter Andreas Steen

Norway

Jakob Stensballe

Denmark

Kristian Strand

Norway

Lovisa Strömmer

Sweden

Kjetil Sunde

Norway

Geir Arne Sunde

Norway

Bjorn-Ove Suserud

Sweden

Leif Svensson

Sweden

Colman Taylor

Australia

Susan Taylor

United States of America

Peter Temesvari

Hungary

Karl-Christian Thies

United Kingdom

Stephen Thomas

United States of America

Julian Thompson

United Kingdom

Kenneth Thorsen

Norway

Chris Thrasher

Canada

Areti Tillou

United States of America

Jens Tingleff

Denmark

Else Tonnesen

Denmark

Karen Tracy

United States of America

Oddvar Uleberg

Norway

Johan Unden

Sweden

Amy Valderrama

United States of America

George Velmahos

United States of America

Wolfgang Voelckel

Austria

Jyrki Vuola

Finland

Henry Wang

United States of America

Roger Watson

United Kingdom

Volker Wenzel

Austria

Knut Wester

Norway

Joanne White

United Kingdom

Torben Wisborg

Norway

Thomas Wurmb

Germany

Jari Yli-Hietanen

Finland

Jackie Younker

United Kingdom

Haixiang Yu

United Kingdom

Peter Zed

Canada

Mariska Zwartsenburg

Netherlands

